# PReS-FINAL-2183: IL-6 amplifies toll like receptor mediated cytokine and chemokine production: implications for the pathogenesis of rheumatic inflammatory diseases

**DOI:** 10.1186/1546-0096-11-S2-O18

**Published:** 2013-12-05

**Authors:** I Caiello, G Minnone, D Holzinger, T Vogl, F De Benedetti, R Strippoli

**Affiliations:** 1 Division of Rheumatology, IRCCS Ospedale Pediatrico Bambino Gesú, Rome, Italy; 2 Department of Paediatric Rheumatology and Immunology, University Children's Hospital, Muenster, Germany; 3 Institute of Immunology, University Hospital Muenster, Muenster, Germany; 4 Vascular Biology and Inflammation, Centro Nacional de Investigaciones Cardiovasculares (CNIC), Madrid, Spain

## Introduction

Interleukin-6 (IL-6) is a pleiotropic cytokine with multiple functions in different pathophysiologic systems. A vast body of evidence suggests a role for IL-6 in rheumatoid arthritis (RA) and in systemic juvenile idiopathic arthritis (JIA). Toll-like receptors (TLRs) are a family of transmembrane glycoproteins with conserved extracellular domains and a cytoplasmic signaling domain homologous to that of IL-1R, called the Toll/IL-1R domain. They bind a wide array of bacterial and viral, as well as endogenous, peptides. They mediate inflammatory cytokine and chemokine secretion, thus controlling the response to pathogens and playing a role in the pathogenesis of inflammatory diseases.

## Objectives

To evaluate whether the exposure to IL-6 affects TLR ligand-induced production of inflammatory cytokines and chemokines in human blood and signalling pathways involved in mononuclear cells (PBMCs), adherent mononuclear cells from synovial fluid of JIA patients (adherent SFMCs) and fibroblast-like synoviocytes from rheumatoid arthritis patients (RA synoviocytes).

## Methods

PBMCs were left untreated or treated with IL-6/sIL-6R and then stimulated with LPS, S100A8, Pam2CSK4, poly(I-C), CpG, MDP, IL-1β. Inflammatory cytokine and chemokine expression was measured by ELISA. Activation of p65 NF-κB was evaluated by Western blot. SFMCs and RA synoviocytes were pretreated with IL-6/sIL-6R or sIL-6R, alone or in combination with Tocilizumab (TCZ), and then stimulated as above.

## Results

Addition of IL-6 to PBMCs stimulation with poly(I-C), CpG, Pam2CSK4, and MDP induced a significant increase of IL-1β and CXCL8 production compared with TLR ligands alone. This was associated with an increase in NF-κB activation. In contrast, addition of IL-6 to PBMCs stimulated with LPS or S100A8 (both ligands of TLR-4) led to reduction of IL-1β and CXCL8 production with reduced NF-κB activation. In PBMCs, IL-6 alone was able to induce IL-1β production in the presence of secondary stimulus (ATP), whereas IL-6/IL-1β co-stimulation led to an increase of CXCL8 and CCL2 production. Differently from PBMCs, addition of IL-6 to adherent SFMCs stimulated LPS or S100A8 led to increased CXCL8, CCL2, and IL-1β expression. Treatment of RA synoviocytes with sIL-6R alone (in the presence of autocrine IL-6 production) led to increased CXCL8 and CCL2 production with concomitant increase of STAT3 and NF-κB phosphorylation. Treatment with sIL-6R also increased IL-1β and LPS induced cytokine and chemokine production. All these effects were neutralized by addition of TCZ.

## Conclusion

Our results point to a role of IL-6 in the amplification of TLR induced inflammatory response both in mononuclear cells and in RA synoviocytes. This effect may be relevant in conditions where high levels of IL-6 are produced in the presence of sIL-6R, such as in joints of patients with rheumatic diseases. Interestingly, we found that the well-known inhibitory effect of IL-6 on TLR4 induced cytokine and chemokine production is specific for peripheral blood cells, while in cells form inflammatory sites (adherent SFMCs or RA synoviocytes) IL-6 synergizes with TLR4 ligands.

## Disclosure of interest

None declared.

